# 400. Rehospitalization of Patients for Recurrent *Clostridioides difficile* Infection

**DOI:** 10.1093/ofid/ofac492.478

**Published:** 2022-12-15

**Authors:** Emily Drwiega, Stuart Johnson, Andrew M Skinner

**Affiliations:** University of Illinois at Chicago, Chicago, Illinois; Hines VA Hospital and Loyola University Medical Center, Hines, Illinois; Loyola University Medical Center, Maywood, Illinois

## Abstract

**Background:**

*Clostridioides difficile* infection (CDI) is an important cause of morbidity and mortality, especially in patients with recurrent episodes and those requiring re-hospitalization. Decreasing recurrent CDI (rCDI) and re-hospitalization for rCDI are important outcomes. The purpose of our study was to evaluate the impact of specialty care from an infectious disease (ID) or gastroenterology (GI) provider, either during the admission of the primary episode or in a follow-up appointment within 30 days.

**Methods:**

This was a single-center, retrospective, chart review at a tertiary medical center. All hospitalized patients with a positive stool test for *C. difficile* (CD) (GI panel PCR, FilmArray, Biofire, or CD PCR, Xpert CD assay, Cepheid) with or without an ICD-10 code Enterocolitis due to CD (A04.7, A04.71, A04.72) from January 2018 through December 2018 were reviewed. Demographic and clinical data at the time of diagnosis and up to 90 days after discharge were collected.

**Results:**

Our criteria identified 602 patients. We excluded 27 patients who died while hospitalized and 104 patients classified as colonized from analysis. Most patients (410/471, 87%) were determined to have a primary CDI episode. After discharge, 57 (14%) patients developed rCDI and 40 patients (10%) died within 90 days of discharge. Among those with rCDI, 40% (23/57) of patients had to be re-hospitalized due to their recurrence. Those re-hospitalized for rCDI developed their recurrence sooner (40% within 45 days, p < 0.005) compared to patients with rCDI that were not re-hospitalized (Figure). ID/GI evaluated 173 of 471 (37%) patients during the index hospitalization or on hospital follow-up. Among patients evaluated by ID/GI, 10.4% had rCDI compared to the 13.8% with no ID/GI intervention. (p=NS). Only 3.5% (6/173) of patients evaluated by ID/GI were re-hospitalized vs 5.7% (17/298) of patients without ID/GI evaluation. (p=NS)

Time to CDI recurrence in re-hospitalized patients

**Figure:**
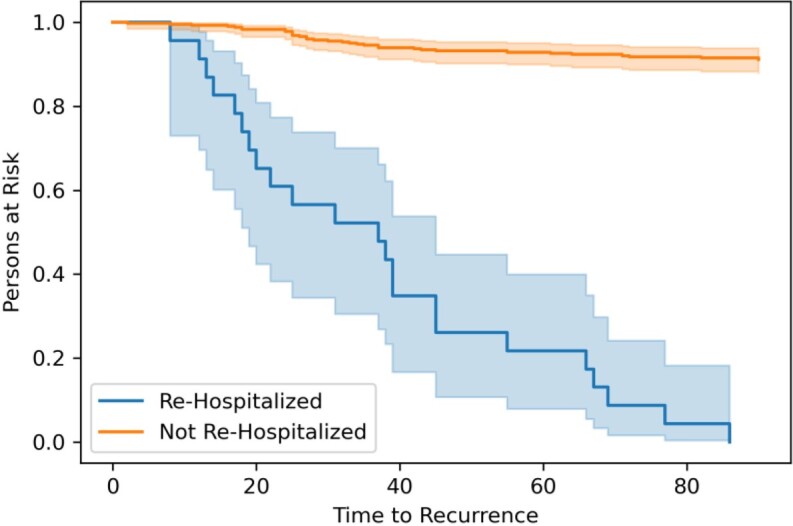

**Conclusion:**

In our study, 40% of patients who had rCDI had to be readmitted for their rCDI. Recurrence occurred significantly earlier in patients who were re-hospitalized. Patients evaluated by ID or GI had a decreased rate of re-hospitalization. Inpatient consultation and timely follow up of hospitalized CDI patients should be studied prospectively to determine the impact on rCDI re-hospitalization.

**Disclosures:**

**Stuart Johnson, M.D.**, Ferring Pharmaceuticals: Membership on Ferring Publication Steering Committee|Ferring Pharmaceuticals: Employee|Summit Plc: Advisor/Consultant.

